# Survival Outcomes of First-Line Therapy in De Novo Metastatic Urothelial Carcinoma with Histologic Subtypes: A National Cancer Database Analysis

**DOI:** 10.3390/cancers18060950

**Published:** 2026-03-14

**Authors:** Zin W. Myint, Feitong Lei, Emma Tay, Bin Huang

**Affiliations:** 1 Division of Medical Oncology, Department of Internal Medicine, University of Kentucky, Lexington, KY 40536, USA; 2 Markey Cancer Center, University of Kentucky, Lexington, KY 40536, USA; 3 Division of Cancer Biostatistics, College of Medicine, University of Kentucky, Lexington, KY 40536, USA; feitonglei0504@uky.edu (F.L.);; 4 Kentucky Cancer Registry, Lexington, KY 40536, USA; 5 Department of Health Sciences, Boston University, Boston, MA 02215, USA; emma.tayy15@gmail.com

**Keywords:** urothelial carcinoma with histologic subtypes, concurrent chemoimmunotherapy, chemotherapy, immunotherapy, national cancer database

## Abstract

**Background**: Urothelial carcinoma with histologic subtypes represent an aggressive and understudied population frequently excluded from prospective clinical trials. Evidence guiding optimal first-line systemic therapy in de novo metastatic disease with histologic subtypes remains limited. **Method**: Patients with de novo stage IV urothelial carcinoma with histologic subtypes diagnosed between 2016 and 2021 were identified from the National Cancer Database (NCDB). Eligible patients received first-line chemotherapy, immunotherapy, or concurrent chemoimmunotherapy (both modalities initiated within 30 days). Overall survival (OS) was compared across groups using Kaplan–Meier methods and compared using log-rank tests and multivariable Cox regression. **Result**: Among 800 patients, 596 (74.5%) received chemotherapy, 106 (13%) received immunotherapy, and 98 (12%) received concurrent chemoimmunotherapy. Survival differed significantly across treatment groups (log-rank *p* = 0.005). Concurrent chemoimmunotherapy was associated with longer OS compared with chemotherapy alone (HR 0.73, 95% CI 0.55–0.93) and immunotherapy monotherapy. Immunotherapy alone was associated with inferior survival compared with chemotherapy. **Conclusions**: In de novo metastatic urothelial carcinoma with histologic subtypes, concurrent chemoimmunotherapy and chemotherapy were associated with superior survival compared with immunotherapy monotherapy. These findings support the consideration of early combination-based strategies and highlight the need for prospective studies dedicated to this high-risk population.

## Simple Summary

Urothelial carcinoma can contain different histologic subtypes, often referred to as variant histologies, which are associated with aggressive behavior and poor outcomes. Patients with these tumors are frequently excluded from clinical trials, leaving limited evidence to guide first-line treatment decisions when the metastatic disease is present at diagnosis. Using a large national cancer database, we examined survival outcomes among patients with de novo metastatic urothelial carcinoma with histologic subtypes treated with chemotherapy, immunotherapy, or a combination chemoimmunotherapy. We found that patients who received combined chemoimmunotherapy had the best survival compared with chemotherapy alone, whereas immunotherapy alone was associated with the poorest outcomes. These findings suggest that immunotherapy by itself may not be sufficient for many patients with histologic subtypes and support the early use of combination-based treatment strategies. Prospective studies focused on this high-risk population are needed to confirm these observations.

## 1. Introduction

Urothelial carcinoma of the bladder demonstrates marked histologic heterogeneity, characterized by a spectrum of non-conventional morphologic patterns that have historically been described as variant histologies and are currently classified as histologic subtypes according to the 2022 World Health Organization criteria [1]. These subtypes are most often identified in association with conventional urothelial carcinoma, whereas tumors composed entirely of a single subtype are infrequently encountered. Overall, histologic subtypes or variant features are reported in approximately 5–25% of bladder cancers, although individual subtypes occur rarely [2,3,4,5]. Squamous differentiation is the most common subtype, accounting for approximately 5% of bladder cancers in Western populations, followed by adenocarcinoma (0.5–2%) and neuroendocrine (small cell) carcinoma (0.5–1%) [2,3,4,5]. Other urothelial subtypes, including micropapillary (0.6–6%), plasmacytoid (1–3%), nested (<1%), and sarcomatoid or carcinosarcoma (0.1–0.3%) variants, are individually uncommon but clinically significant [2,3,4,5].

Beyond morphologic diversity, urothelial carcinoma with histologic subtypes often demonstrates distinct clinical behavior compared with conventional urothelial carcinoma. These tumors are frequently associated with advanced stage at presentation, aggressive disease progression and poorer overall prognosis. In clinical practice, the presence of variant histology can influence both diagnostic evaluation and treatment decision-making. However, despite their clinical importance, variant histologies remain underrepresented in many prospective clinical trials evaluating systemic therapies for advanced urothelial carcinoma. Consequently, treatment recommendations for metastatic disease are often extrapolated from studies conducted primarily in patients with conventional urothelial carcinoma. Real-world population-based analyses therefore provide an important opportunity to better characterize treatment patterns and survival outcomes among patients with metastatic urothelial carcinoma harboring variant histology.

Patients with histologic subtypes are frequently excluded from prospective clinical trials and are underrepresented in randomized studies guiding first-line systemic therapy for metastatic disease. Consequently, evidence guiding outcomes with first-line chemotherapy, immunotherapy, or combination approaches in metastatic urothelial carcinoma with histologic subtypes is largely derived from retrospective real-world cohorts, with limited subtype-specific data and heterogeneous treatment populations [6,7,8]. Available real-world studies suggest that patients with histologic subtypes experience inferior survival outcomes compared with conventional urothelial carcinoma when treated with first-line systemic therapy, despite variable initial responses [6,7,8]. Given the rarity of individual histologic subtypes and their frequent coexistence with conventional urothelial carcinoma, large national registry datasets provide a valuable opportunity to evaluate treatment patterns and outcomes across diverse patient populations in real-world clinical practice.

To address this gap, we analyzed the National Cancer Database (NCDB) to compare real-world outcomes of patients with de novo metastatic histologic subtypes of urothelial carcinoma treated with first-line chemotherapy, immunotherapy or concurrent chemoimmunotherapy.

## 2. Patients and Methods

### 2.1. Data Source

We used data from the NCDB, a national oncology registry jointly sponsored by the American College of Surgeons and the American Cancer Society. The NCDB contains de-identified patient information reported by hospital-based cancer registries from more than 1500 Commission on cancer-accredited institutions and captures approximately 70% of newly diagnosed cancer cases across the United States [9,10,11]. The NCDB maintains a large longitudinal repository, currently comprising over 34 million cancer records. Data included in the NCDB are collected from participating institutions using standardized data abstraction procedures and coding definitions established by the Commission on Cancer. The NCDB has been widely used for observational oncology research to evaluate treatment patterns, disparities in cancer care, and survival outcomes across large national populations. Its large sample size makes it particularly useful for studying relatively uncommon clinical scenarios, including variant histology subtypes of urothelial carcinoma, which are often underrepresented in prospective clinical trials. In accordance with institutional requirements, this study received a waiver of Institutional Review Board (IRB) review.

### 2.2. Study Population

Patients with de novo metastatic urothelial carcinoma were identified from the NCDB participant user data file. Eligible primary sites included the bladder (ICD-O-3 topography codes C67.0–C67.9), renal pelvis (C65.0), and ureter (C66.9). The study was restricted to adults (age ≥ 18 years) with metastatic urothelial carcinoma as the first primary cancer diagnosed between 2016 and 2021. Patients who underwent first-line surgery, had unknown chemotherapy or immunotherapy status, or initiated chemotherapy or radiotherapy more than 180 days after diagnosis were excluded.

To focus on histology subtypes, patients with pure urothelial carcinoma (ICD-O-3 morphology codes 8120, 8123, 8130, 8133) were excluded. Variant histology subtypes were classified according to WHO 2016 criteria [12] and included squamous cell carcinoma (8070, 8071, 8073, 8050, 8052, 8053), glandular differentiation/adenocarcinoma (8140, 8143, 8260, 8490), micropapillary urothelial carcinoma (8131, 8133), sarcomatoid carcinoma (8122, 8123), and small cell/neuroendocrine carcinoma (8041, 8043, 8045, 8243, 8246). Variant histology patterns frequently occur in combination with conventional urothelial carcinoma rather than as pure histologic entities. However, large administrative and registry-based datasets typically classify tumors according to the predominant histologic subtype reported by the treating institution. As a result, the present analysis reflects the dominant histologic classification recorded in the NCDB rather than the proportion of variant differentiation within individual tumors. While this limitation restricts the ability to evaluate outcomes according to the percentage of variant histology present, the use of standardized ICD-O morphology codes provides a consistent and reproducible method for identifying histologic subtypes across large national datasets.

### 2.3. Treatment Groups

Patients were stratified according to first-line systemic therapy, defined by the type and timing of chemotherapy and immunotherapy initiated within 180 days of diagnosis. Three primary treatment groups were defined: (1) chemotherapy alone (C), patients who received chemotherapy without immunotherapy; (2) immunotherapy alone (I), patients who received immunotherapy without chemotherapy; and (3) concurrent chemoimmunotherapy (CI), patients who received both agents with initiation dates within 30 days of one another, with at least one agent initiated within 180 days of diagnosis.

### 2.4. Covariates

Patient demographics and tumor characteristics were examined, including age (40–65 years, 66–79 years, and ≥80 years), sex, race/ethnicity (White, Black, other), primary cancer site, tumor grade, comorbidity burden (Charlson comorbidity index: 0, 1, and ≥2), insurance status, facility type (academic vs. non-academic), metropolitan vs. urban classification, median household income (ZIP-code-level quartile), high school education attainment (ZIP-code-level quartile), and distance to the treating facility (straight-line distance in miles).

### 2.5. Statistical Analysis

Baseline demographic and clinical characteristics across treatment groups were compared using chi-square tests for categorical variables. Descriptive statistics were used to summarize baseline demographic and clinical characteristics across treatment groups. Continuous variables were reported as medians with interquartile ranges, and categorical variables were summarized using frequencies and percentages. Comparisons between treatment groups were performed using the chi-square test for categorical variables. A secondary descriptive analysis characterized the distribution of time from diagnosis to treatment initiation and among patients receiving both agents, the interval between chemotherapy and immunotherapy initiation.

The primary endpoint was overall survival (OS), defined as the time from initiation of first-line systemic therapy to death or last follow-up. For patients receiving concurrent chemoimmunotherapy, the index date was defined as the initiation of the latter of the two systemic agents. This approach was prespecified to minimize immortal time bias, as patients must survive long enough to receive both agents to be classified in the concurrent treatment group. OS was estimated using the Kaplan–Meier method, with log-rank tests used to compare survival differences among treatment groups. Multivariable Cox Proportional hazards regression with backward stepwise selection was performed to identify the final covariate set and estimate adjusted hazard ratios for OS. In the multivariable Cox regression model, clinically relevant covariates including age group and year of diagnosis were retained irrespective of statistical significance to account for potential confounding related to frailty and temporal treatment evolution. All statistical tests were two-sided, and a *p*-value < 0.05 was considered statistically significant. Analyses were conducted using SAS ^®^ version 9.4 (SAS Institute Inc., Cary, NC, USA)

## 3. Results

Among the 800 patients included, chemotherapy alone was the most common first-line regimen (*n* = 596, 74.5%), followed by immunotherapy alone (*n* = 106, 13%) and concurrent chemoimmunotherapy (CI; *n* = 98, 12%) (Table 1). The bladder was the predominant primary tumor site (92%). Approximately two-thirds of patients were male, and 86% were White (Table 1). The distribution of individual histologic subtypes across treatment groups is detailed in Supplementary Table S1. Overall, the distribution of histologic subtypes demonstrated substantial heterogeneity within the cohort. Squamous differentiation represented the most frequently observed subtype, followed by adenocarcinoma and neuroendocrine carcinoma. Less common variants such as micropapillary, plasmacytoid, nested, and sarcomatoid histologies were present in smaller proportions but collectively represented a clinically important subgroup. The relative distribution of histologic subtypes across treatment groups was generally similar, although certain aggressive subtypes appeared more frequently in patients receiving combination systemic therapy. These differences may reflect physician treatment selection patterns in routine clinical practice.

Patients treated with immunotherapy alone were older, with 50% aged 80 years or older, compared with 20% in the chemotherapy only group and 30% in the CI group. These differences likely reflect real-world treatment selection patterns. Older patients and those with greater comorbidity burden may be less likely to receive cytotoxic chemotherapy due to concerns regarding treatment tolerability. Consequently, immunotherapy monotherapy may be preferentially used in patients who are not candidates for platinum-based chemotherapy. In contrast, patients receiving concurrent chemoimmunotherapy may represent individuals with better functional status who are eligible for more intensive treatment strategies. The increasing use of immunotherapy and combination therapy in later years of the study period likely reflects evolving treatment paradigms following the introduction of immunotherapy into routine clinical practice. Patients receiving immunotherapy alone were also more likely to be female (46%) compared with those receiving chemotherapy alone or CI (both 30%). The proportion of patients receiving immunotherapy alone or concurrent CI increased substantially in later diagnosis years. Among patients diagnosed in 2020, immunotherapy-only and CI groups comprised 28.3% and 30.6% of cases, respectively, compared with 6.6% and 0% among those diagnosed in 2016 (*p* < 0.001).

The median time from cancer diagnosis to treatment initiation was 35 days (interquartile range [IQR], 22–57 days) for chemotherapy alone, 51 days (IQR, 30–82 days) for immunotherapy alone, and 41 days (IQR, 29–63 days) for CI, measured from diagnosis to initiation of the latter treatment. Among patients receiving CI, the median interval between chemotherapy and immunotherapy initiation was 0 days (IQR, 0–0 days), indicating that both agents were initiated on the same day in most cases.

### 3.1. Histologic Subtype Distribution

The distribution of histologic subtypes differed significantly across treatment groups (*p* < 0.001). Overall, small-cell/neuroendocrine carcinoma comprised 47.5% of cases (*n* = 380), squamous cell carcinoma 20.0% (*n* = 160), adenocarcinoma 15.8% (*n* = 126), sarcomatoid carcinoma 9.8% (*n* = 78), and micropapillary urothelial carcinoma 7.0% (*n* = 56) (Supplementary Table S1). Notably, small-cell/neuroendocrine carcinoma represented 76.5% of patients in the concurrent chemoimmunotherapy group compared with 49.3% in the chemotherapy group and 10.4% in the immunotherapy-only group. In contrast, squamous cell carcinoma and adenocarcinoma were more evenly distributed across treatment groups. These differences in subtype distribution likely reflect variation in tumor histology, disease aggressiveness and treatment selection patterns in routine clinical practice.

### 3.2. Survival Outcomes

**Figure 1:**
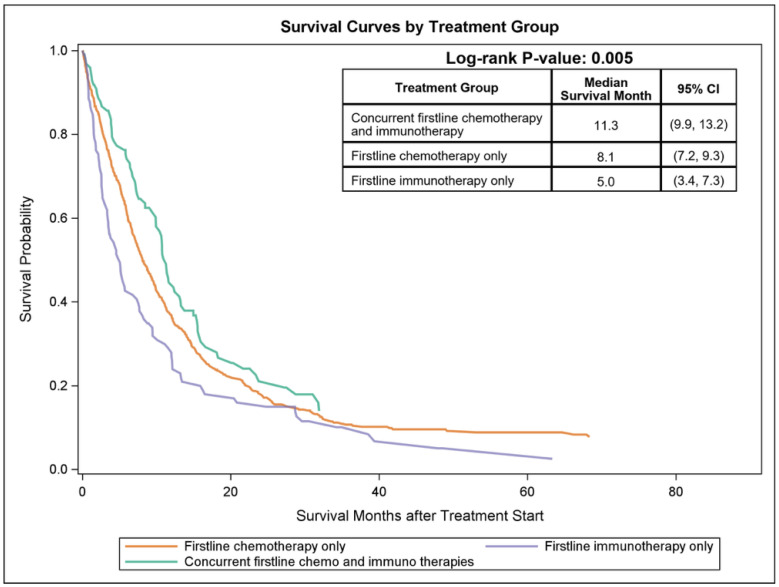
Kaplan–Meier overall survival stratified by treatment groups in de novo metastatic urothelial carcinoma with variant histology subtypes.

Kaplan–Meier survival analysis demonstrated significant differences in OS across the three treatment groups (log-rank, *p* = 0.005) (Figure 1). Patients receiving concurrent CI had the longest median OS at 11.3 months (95% CI, 9.9–13.2), followed by chemotherapy alone at 8.1 months (95% CI, 7.2–9.3) and immunotherapy alone at 5.0 months (95% CI, 3.4–7.3) (Figure 1). The observed differences in survival outcomes across treatment groups highlight the aggressive clinical course of metastatic urothelial carcinoma with variant histologies. Although immunotherapy monotherapy has demonstrated durable responses in selected patients with metastatic urothelial carcinoma, the relatively short median survival observed in the immunotherapy-only group suggests that this strategy may provide limited benefit for many patients with variant histology. Treatment selection in this observational cohort may also reflect underlying differences in patient characteristics, including age, comorbidity burden, and eligibility for cytotoxic chemotherapy. Nevertheless, the survival advantage observed with concurrent chemoimmunotherapy supports the hypothesis that combination treatment strategies may provide improved disease control in this high-risk population.

**Figure 2:**
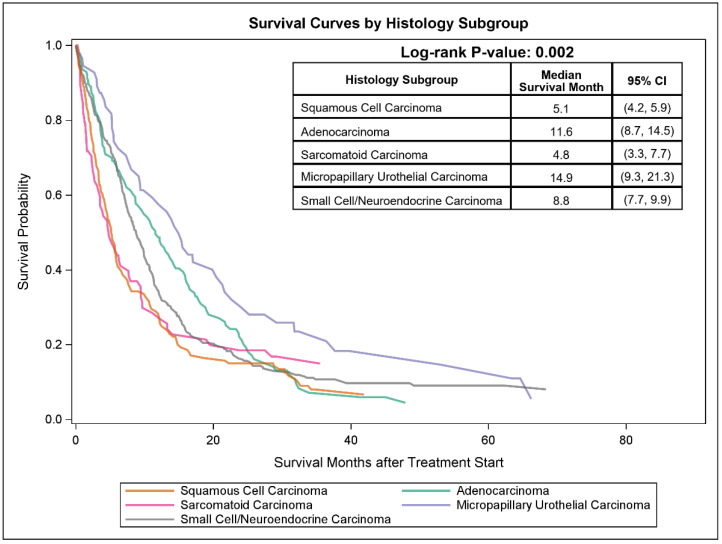
Kaplan–Meier overall survival stratified by histologic subgroups.

Descriptive survival analysis by histologic subtype demonstrated heterogeneity in outcomes. Median OS was 14.9 months (95% CI 9.3–21.3) for micropapillary carcinoma, 11.6 months (95% CI 8.7–14.5) for adenocarcinoma, 8.8 months (95% CI 7.7–9.9) for small cell/neuroendocrine carcinoma, 5.1 months (95% CI 4.2–5.9) for squamous cell carcinoma, and 4.8 months (95% CI 3.3–7.7) for sarcomatoid carcinoma [Figure 2] (Supplementary Table S2). These analyses were descriptive and not powered for subtype-specific comparative effectiveness conclusions.

In multivariable Cox proportional hazards regression including age, year of diagnosis, and histologic subtype, concurrent chemoimmunotherapy was associated with longer OS compared with chemotherapy alone (HR 0.73, 95% CI 0.55–0.93) (Table 2). Immunotherapy monotherapy was not significantly associated with OS after adjustment (HR 1.23; 95% CI 0.97–1.57). A Charlson comorbidity index ≥ 2 independently predicted worse survival (HR 1.69; 95% CI 1.37–2.07). Renal pelvis primary tumors were also associated with inferior OS compared with bladder tumors (HR 1.54; 95% CI 1.08–2.20) (Table 2).

In a sensitivity analysis restricted to patients younger than 80 years (*n* = 600), the overall conclusions remained consistent. Concurrent chemoimmunotherapy was associated with improved survival compared with chemotherapy alone (HR 0.68, 95% CI 0.50–0.94), while immunotherapy monotherapy was associated with inferior survival (HR 1.39; 95% CI 1.03–1.88). These findings suggest that age imbalance alone does not fully account for observed survival differences.

## 4. Discussion

This large NCDB analysis provides real-world evidence on first-line systemic therapy patterns and survival outcomes in patients with de novo metastatic urothelial carcinoma with histologic subtypes. In our study, concurrent chemoimmunotherapy was associated with longer overall survival compared to other treatment groups. These findings highlight that immunotherapy monotherapy may be insufficient for many patients with histologic subtypes and support the consideration of early combination-based strategies when clinically feasible. Importantly, substantial heterogeneity in survival was observed across histologic subtypes. Micropapillary and adenocarcinoma subtypes demonstrated comparatively longer median survival, whereas sarcomatoid and squamous variants were associated with poorer outcomes. These findings are consistent with the prior literature describing distinct biological behavior among variant histologies [6,8,13,14,15,16] and underscore the importance of interpreting population-level treatment associations cautiously. Notably, small cell/neuroendocrine carcinoma was more frequently represented in the concurrent chemoimmunotherapy group, which may partially influence observed treatment associations and further underscores the complexity of interpreting retrospective registry data.

Importantly, prospective randomized trials specifically designed to evaluate concurrent chemoimmunotherapy in urothelial carcinoma with histologic subtypes are lacking, as patients with histologic subtypes have historically been excluded or underrepresented in registrational studies. Consequently, treatment decisions in this population have largely relied on extrapolation from conventional urothelial carcinoma and retrospective real-world evidence. In this context, our findings provide novel population-level data suggesting a potential survival advantage with upfront combination therapy in a high-risk and understudied group.

Our results align with emerging clinical data suggesting heterogeneity of immunotherapy monotherapy activity by histologic composition. The SAUL study was a large multinational phase 3b single-arm trial evaluating single-agent atezolizumab in a real-world population of patients with locally advanced or metastatic urothelial or non-urothelial carcinoma of the urinary tract, including histologic subtypes typically excluded from registrational trials. In this subgroup, antitumor activity was modest with an ORR of 9% and a median OS of 7.3 months, both numerically inferior to outcomes observed in the overall cohort. These findings reinforce concerns regarding the limited efficacy of immunotherapy monotherapy in tumors with the predominant variant or non-urothelial histology [17,18].

Similarly, a multicenter retrospective European study evaluated outcomes in patients with metastatic bladder cancer harboring predominant variant histology (>50%) or non-urothelial carcinoma subtypes, including neuroendocrine, adenocarcinoma, squamous cell carcinoma, and micropapillary variants. The study demonstrated substantially higher response rates with first-line chemotherapy compared with immunotherapy (ORR 62.2% vs. 22%). Platinum-based regimens, including dose-dense MVAC and platinum doublets, achieved the highest response rates, underscoring the continued importance of cytotoxic chemotherapy in this biologically aggressive population [6].

Although not designed to evaluate histologic subtypes, large phase III trials of chemoimmunotherapy in unselected urothelial carcinoma populations provide additional biologic context. In KEYNOTE-361 [19] and IMvigor 130 [20], concurrent chemoimmunotherapy demonstrated improved early disease control compared with immunotherapy monotherapy, despite not meeting all primary endpoints. More recently, the CheckMate 901 trial [21] showed a significant overall survival benefit with nivolumab plus gemcitabine-cisplatin compared with chemotherapy alone, establishing concurrent chemoimmunotherapy as a contemporary first-line standard in unselected patients with advanced urothelial carcinoma. While these studies do not allow subtype-specific conclusions, they support the biologic plausibility of combination approaches in aggressive disease settings where immunotherapy monotherapy has limited activity.

The present study reflects outcomes during a transitional therapeutic era (2016–2021), preceding widespread frontline adoption of antibody–drug–conjugate (ADC)-based combinations such as enfortumab vedotin plus pembrolizumab. Accordingly, our results should be interpreted as representing real-world outcomes in the pre-ADC era. Rather than diminishing relevance, this context provides an important benchmark against which contemporary combination strategies may be evaluated, particularly in variant histology populations that remain underrepresented in prospective trials. The phase III EV-302 trial [22,23] demonstrated a significant survival benefit with enfortumab vedotin plus pembrolizumab compared with platinum-based chemotherapy in previously untreated advanced urothelial carcinoma, establishing antibody–drug–conjugate (ADC)-based combination therapy as a new first-line standard. Although outcomes by histologic subtype were not reported, the consistent benefit observed across the overall population supports the biologic rationale for upfront combination strategies. In the absence of subtype-specific prospective data, these results provide important context for our real-world findings favoring combination-based approaches in de novo metastatic urothelial carcinoma with histologic subtypes. Importantly, variant histology patients remain underrepresented in contemporary ADC trials, and subtype-specific outcomes have not been fully characterized.

Supporting this concept, ADC-based strategies appear to retain activity across several histologic subtypes [24,25,26,27]. A retrospective single-center study evaluated the efficacy of first-line pembrolizumab plus enfortumab vedotin in patients with metastatic urothelial carcinoma across different histological subtypes compared with predominant transitional cell. A total of 71 patients were included: 37 with predominant transitional urothelial carcinoma, 6 with transitional urothelial carcinoma containing <50% squamous or glandular differentiation, 13 with transitional urothelial carcinoma containing >50% squamous or glandular differentiation, 7 with micropapillary variant and 8 with other histologies, including sarcomatoid, plasmacytoid, nested and lipid-rich variants. The overall response rate (ORRs) were 57.6%, 16.6%, 0%, 57.1%, and 25%, respectively [28].

Additional support for ADC-based combination therapy comes from a recent case series in plasmacytoid urothelial carcinoma, a rare and aggressive histologic subtype. Among three patients with stage IV treated with enfortumab vedotin and pembrolizumab, two achieved durable disease stabilization for 8–10 months with manageable toxicity, while one patient experienced early progression [29]. Although limited by small sample size, these findings suggest potential activity of ADC-based combination therapy in select aggressive variant histologies.

Consistently, the UNITE study [30] demonstrated that enfortumab vedotin monotherapy produced responses across multiple histologic subtypes; the ORR were 44% for all patients with histologic subtypes, 50% for those with <50% histologic subtype, 35% for those with >50% histologic subtype, and 14% for those with pure histologic subtype. However, efficacy declined with increasing variant predominance and was particularly limited in pure variant and neuroendocrine histologies. Notably, prospective efforts to address this evidence gap are emerging. A currently ongoing prospective clinical trial (NCT05756569) is evaluating combination systemic therapy strategies specifically in patients with urothelial carcinoma and histologic subtypes. While results are not yet available, this study underscores growing recognition of the unmet need in this population and may provide important prospective validation of combination-based approaches suggested by retrospective and real-world data.

Our study has important strengths, including the use of a large national dataset and adjustment for key clinical covariates. However, several important limitations of this study should be acknowledged. First, as a retrospective observational analysis, this study remains subject to residual confounding and treatment selection bias. The NCDB does not capture performance status (ECOG), and patients receiving immunotherapy monotherapy were older and likely frailer. Although we adjusted for age and comorbidity and performed age-restricted sensitivity analyses demonstrating consistent results, unmeasured confounding cannot be excluded. Additionally, residual confounding related to unmeasured variables such as performance status and metastatic burden cannot be excluded. Second, the NCDB does not capture detailed treatment information, including the specific chemotherapy regimens administered, dosing intensity, or treatment duration, precluding analyses by individual platinum-based or non-platinum regimens. In addition, the database lacks granular pathologic detail regarding the proportion of histologic subtypes (i.e., <50% vs. >50% or pure), which may have biologically and clinically distinct behavior. In addition, registry-based analyses may be subject to potential misclassification of clinical variables due to variations in coding practices across institutions. Although standardized coding guidelines are used, the accuracy of histologic subtype classification ultimately depends on institutional pathology reporting and registry abstraction processes. Furthermore, because the NCDB captures data primarily from Commission on Cancer-accredited institutions, treatment patterns observed in this analysis may not fully reflect those in non-accredited centers. Finally, NCDB does not provide data on radiographic treatment response, or subsequent lines of therapy, limiting interpretation of treatment sequencing and long-term disease control. Molecular and biomarker information, including genomic alterations and PD-L1 expression status are not available.

## 5. Conclusions

In patients with de novo metastatic urothelial carcinoma with histologic subtypes, concurrent chemoimmunotherapy and chemotherapy were associated with superior survival compared with immunotherapy monotherapy. These findings suggest that immunotherapy monotherapy may be insufficient for many patients with histologic subtypes and support the early use of combination-based strategies when clinically feasible. Given the lack of prospective subtype-specific data, our results provide important real-world evidence to inform treatment selection and underscore the need for prospective clinical trials dedicated to urothelial carcinoma with histologic subtypes.

## Figures and Tables

**Table 1:** Baseline characteristics of patients with de novo metastatic urothelial carcinoma with variant histology by treatment group.

	Firstline Chemotherapy Only	Firstline Immunotherapy Only	Concurrent Firstline Chemo- and Immunotherapies	Total	*p*-Value
	(N = 596)	(N = 106)	(N = 98)	(N = 800)	
**Primary Site**, n (%)					0.00861
Bladder	557 (93.5%)	88 (83.0%)	90 (91.8%)	735 (91.9%)	
Renal pelvis	21 (3.5%)	11 (10.4%)	5 (5.1%)	37 (4.6%)	
Ureter	18 (3.0%)	7 (6.6%)	3 (3.1%)	28 (3.5%)	
**Age Group**, n (%)					<0.0001 ^1^
18–39	17 (2.9%)	0 (0.0%)	2 (2.0%)	19 (2.4%)	
40–65	256 (43.0%)	28 (26.4%)	28 (28.6%)	312 (39.0%)	
66–79	204 (34.2%)	26 (24.5%)	39 (39.8%)	269 (33.6%)	
≥80	119 (20.0%)	52 (49.1%)	29 (29.6%)	200 (25.0%)	
**Race**, n (%)					0.72081
White	506 (84.9%)	92 (86.8%)	89 (90.8%)	687 (85.9%)	
Black	66 (11.1%)	9 (8.5%)	6 (6.1%)	81 (10.1%)	
Other	19 (3.2%)	4 (3.8%)	3 (3.1%)	26 (3.3%)	
Unknown	5 (0.8%)	1 (0.9%)	0 (0.0%)	6 (0.8%)	
**Sex**, n (%)					0.00651
Male	413 (69.3%)	57 (53.8%)	68 (69.4%)	538 (67.3%)	
Female	183 (30.7%)	49 (46.2%)	30 (30.6%)	262 (32.8%)	
**Metro**, n (%)					0.71871
Non-Metro	99 (16.6%)	23 (21.7%)	16 (16.3%)	138 (17.3%)	
Metro	480 (80.5%)	81 (76.4%)	80 (81.6%)	641 (80.1%)	
Unknown	17 (2.9%)	2 (1.9%)	2 (2.0%)	21 (2.6%)	
**Zip-level Median Household Income**, n (%)					0.55711
<$40,227	90 (15.1%)	15 (14.2%)	11 (11.2%)	116 (14.5%)	
$40,227–$50,353	100 (16.8%)	23 (21.7%)	15 (15.3%)	138 (17.3%)	
$50,354–$63,332	141 (23.7%)	21 (19.8%)	24 (24.5%)	186 (23.3%)	
63,333+	170 (28.5%)	25 (23.6%)	34 (34.7%)	229 (28.6%)	
Unknown	95 (15.9%)	22 (20.8%)	14 (14.3%)	131 (16.4%)	
**Zip-level High School Education attainment**, n (%)					0.13581
≤82.4%	88 (14.8%)	23 (21.7%)	13 (13.3%)	124 (15.5%)	
82.5–89.1%	143 (24.0%)	17 (16.0%)	17 (17.3%)	177 (22.1%)	
89.2–93.7%	154 (25.8%)	23 (21.7%)	28 (28.6%)	205 (25.6%)	
>93.7	118 (19.8%)	21 (19.8%)	27 (27.6%)	166 (20.8%)	
Unknown	93 (15.6%)	22 (20.8%)	13 (13.3%)	128 (16.0%)	
**Insurance**, n (%)					0.00271
Not Insured	14 (2.3%)	2 (1.9%)	2 (2.0%)	18 (2.3%)	
Private Insurance	183 (30.7%)	14 (13.2%)	28 (28.6%)	225 (28.1%)	
Medicaid	83 (13.9%)	11 (10.4%)	6 (6.1%)	100 (12.5%)	
Medicare	311 (52.2%)	78 (73.6%)	62 (63.3%)	451 (56.4%)	
Unknown	5 (0.8%)	1 (0.9%)	0 (0.0%)	6 (0.8%)	
**Academic Facility**, n (%)					0.66891
No	384 (64.4%)	73 (68.9%)	63 (64.3%)	520 (65.0%)	
Yes	212 (35.6%)	33 (31.1%)	35 (35.7%)	280 (35.0%)	
**Charlson comorbidity index**, n (%)					0.16951
0	398 (66.8%)	60 (56.6%)	62 (63.3%)	520 (65.0%)	
1	105 (17.6%)	21 (19.8%)	15 (15.3%)	141 (17.6%)	
≥2	93 (15.6%)	25 (23.6%)	21 (21.4%)	139 (17.4%)	
**Grade**, n (%)					<0.0001 ^1^
Low	20 (3.4%)	4 (3.8%)	2 (2.0%)	26 (3.3%)	
High	170 (28.5%)	17 (16.0%)	6 (6.1%)	193 (24.1%)	
N/A	406 (68.1%)	85 (80.2%)	90 (91.8%)	581 (72.6%)	
**Distance Between Patient’s Residence and Hospital**, n (%)					0.72361
<10 Miles	230 (38.6%)	43 (40.6%)	36 (36.7%)	309 (38.6%)	
10–50 Miles	231 (38.8%)	32 (30.2%)	38 (38.8%)	301 (37.6%)	
50–100 Miles	29 (4.9%)	5 (4.7%)	7 (7.1%)	41 (5.1%)	
100+ Miles	21 (3.5%)	5 (4.7%)	4 (4.1%)	30 (3.8%)	
Unknown	85 (14.3%)	21 (19.8%)	13 (13.3%)	119 (14.9%)	
**Alive**, n (%)					0.02071
No	505 (84.7%)	96 (90.6%)	75 (76.5%)	676 (84.5%)	
Yes	91 (15.3%)	10 (9.4%)	23 (23.5%)	124 (15.5%)	
**Year of Diagnosis**, n (%)					<0.0001 ^1^
2016	145 (24.3%)	7 (6.6%)	0 (0.0%)	152 (19.0%)	
2017	104 (17.4%)	16 (15.1%)	4 (4.1%)	124 (15.5%)	
2018	107 (18.0%)	12 (11.3%)	4 (4.1%)	123 (15.4%)	
2019	86 (14.4%)	21 (19.8%)	22 (22.4%)	129 (16.1%)	
2020	74 (12.4%)	30 (28.3%)	30 (30.6%)	134 (16.8%)	
2021	80 (13.4%)	20 (18.9%)	38 (38.8%)	138 (17.3%)	

^1^ Chi-Square *p*-value.

**Table 2:** Univariate and multivariable Cox regression analysis of overall survival.

	Univariate HR (95% CI)	*p*-Value	Multivariable HR (95% CI)	*p*-Value
**Treatment group**		0.005		0.007
Chemotherapy (reference)				
Immunotherapy	1.32 (1.06–1.64)	0.019	1.23 (0.97–1.57)	
Concurrent Chemoimmunotherapy	0.80 (0.63–1.02)	0.042	0.77 (0.6–0.98)	
**Age group**		0.421		0.267
40–65 (reference)	-	-	-	-
18–39	0.80 (0.48–1.33)	0.39	0.87 (0.52–1.46)	0.60
66–79	0.87 (0.73–1.04)	0.13	0.84 (0.70–1.01)	0.064
**Charlson Comorbidity Index**		<0.001		<0.001
0 (reference)				
1	1.02 (0.83–1.25)	0.27	0.998 (0.81–1.23)	
≥2	1.50 (1.24–1.84)	<0.001	1.69 (1.37–2.07)	
**Primary site**		0.004		0.046
Bladder (reference)				
Renal pelvis	1.75 (1.24–2.47)	0.002	1.54 (1.08–2.20)	0.018
Ureter	1.19 (0.79–1.81)	0.49	1.19 (0.79–1.81)	0.40
**Year of Diagnosis**		0.472		0.106
2016 (reference)	-	-	-	-
2017	0.96 (0.75–1.24)	0.76	1.00 (0.78–1.30)	0.99
2018	1.04 (0.81–1.34)	0.75	1.11 (0.86–1.44)	0.43
2019	1.14 (0.89–1.46)	0.30	1.33 (1.03–1.73)	0.029
2020	0.87 (0.67–1.12)	0.28	0.93 (0.71–1.22)	0.61
2021	1.05 (0.81–1.36)	0.72	1.18 (0.90–1.54)	0.23
**Histologic Subtype**		0.002		<0.001
Adenocarcinoma (reference)	-	-	-	-
Micropapillary	0.76 (0.54–1.08)	0.13	0.64 (0.45–0.92)	0.016
Sarcomatoid	1.26 (0.92–1.72)	0.15	1.16 (0.84–1.59)	0.37
Small cell/neuroendocrine	1.11 (0.89–1.38)	0.36	1.17 (0.93–1.48)	0.18
Squamous cell	1.42 (1.10–1.83)	0.007	1.34 (1.03–1.74)	0.028

## Data Availability

The data presented in this study are available on request from the corresponding author.

## Supplementary Materials

The following supporting information can be downloaded at https://www.mdpi.com/article/10.3390/cancers18060950/s1. Table S1: Distribution of Histologic Subtypes by Treatment Group; Table S2: Median Overall Survival by Histologic Subtype.
