# Supplementary material for: Survival Outcomes of First-Line Therapy in De Novo Metastatic Urothelial Carcinoma with Histologic Subtypes: A National Cancer Database Analysis
Source: Cancers (Basel). 2026 Mar 14;18(6):950. doi: 10.3390/cancers18060950 (PMC13025140; doi:10.3390/cancers18060950)
Supplement: Supplementary file 1 [file cancers-18-00950-s001.zip › cancers-4167129-supplementary.pdf]

Supplementary Table S1: Distribution of Histologic Subtypes by Treatment Group

Histologic Subtype	Chemotherapy (n=596)	Immunotherapy (n=106)	Concurrent Chemoimmunotherapy (n=98)	Total (n=800)	p-value
Squamous	107 (18%)	42 (39.6%)	11 (11.2%)	160 (20%)	<0.001
Adenocarcinoma	102 (17.1%)	19 (17.9%)	5 (5.1%)	126 (15.8%)	
Sarcomatoid	51 (8.6%)	22 (20.8%)	5 (5.1%)	78 (9.8%)	
Micropapillary	42 (7.0%)	12 (11.3%)	2 (2%)	56 (7%)	
Small cell/neuroendocrine	294 (49.3%)	11 (10.4%)	75 (76.5%)	380 (47.5%)	

Supplementary Table S2: Median Overall Survival by Histologic Subtype

Subtype	Number	Events	Median overall survival (months)	95% CI	Subtype
Adenocarcinoma	126	105	11.6	8.7-14.5	Adenocarcinoma
Micropapillary	56	45	14.9	9.3-21.3	Micropapillary
Sarcomatoid	78	63	4.8	3.3-7.7	Sarcomatoid
Small cell	380	323	8.8	7.7-9.9	Small cell
Squamous	160	140	5.1	4.2-5.9	Squamous
